# Carcinogenic Activity of Lipoid Substances

**DOI:** 10.1038/bjc.1949.15

**Published:** 1949-03

**Authors:** I. Hieger

## Abstract

**Images:**


					
CARCINOGENMC ACTIVITY          OF LIPOID SUBSTANCES.

I. HIEGER.

From the Chester Beatty Research Institute, Royal Cancer Hospital, London, S.W. 3.

Received for publication January 18, 1949.

THE investigation of carcinogenic substances which have been obtained
from human tissues (Kleinenberg, Neufach and Shabad, 1940) and from other
biological sources has been the subject of earlier publications from this Institute
(Hieger, 1940, 1941, 1946, 1947), reporting that:

1. A carcinogen is present in extracts from human subjects who have
died of cancer or of other causes.

2. The carcinogen is found in the unsaponifiable part of a number of
tissues, such as liver, lung, kidney and muscle.

3. It is found in the cholesterol-rich part of the unsaponifiable fraction,
which contains c. 85 per cent of the digitonin precipitable sterol and is
referred to below as "Fraction A" or "Stage A," or "- 10? fraction,"
since it crystallizes out at - 10? when the unsaponifiable fraction of the
tissue (doubly saponified) is crystallized from methyl alcohol.

4. Commercial cholesterol containing 95-98 per cent of the pure sterol
is carcinogenic, and its potency is of the same order as that of the active
fractions from human tissues.

With very few exceptions the carcinogen in biological material is slow acting
(Table V), by comparison with synthetic carcinogens. Hence the tests on
animals are lengthy.

Since some of the experiments described here were in progress when the papers
referred to above were published and others have not before been described in
detail, a certain amount of repetition is unavoidable.

I. HIEGER

1. Technique used in these experiments for testing for carcinogenic activity.

Unless otherwise stated, the substance is injected subeutem. (15 per cent
solution in lard) into mice of C57 strain or of mixed commercial stock. The
injection of 01-01-4 ml. is repeated fortnightly until a reservoir remains under
the skin; injections are suspended as long as a nodule can be detected by touch.
All tumours referred to are spindle-cell sarcomas arising at the site of injection
(Fig. 1-8). Precautions were taken to prevent contamination by carcinogens
from outside (Hieger, 1946).

2. Control experiment on reagents used in the saponification of tissue by potassium

hydroxide and alcohol.

Since the great majority of the carcinogenic fractions had undergone saponi-
fication at least once, control experiments on the process itself acting on non-
carcinogenic tissue components were made. The saponification conditions were
described in detail (Hieger, 1946); about 1500 g. tissue is refluxed on the water
bath with 400 g. KOH and 500 c.c. distilled alcohol for 2-3 hours and then
extracted several times with ether. Carcinogens might be formed during
saponification from precursors in the tissues or from the reagents themselves.

In the earlier experiments the alcohol was industrial spirit (the manufacturers
state that it consists of ethyl and methyl alcohols in the ratio of 19:1 with 5 per
cent water), which in separate experiments was found to darken rapidly on
refluxing with KOH owing to resinous condensation of aldehyde. Acetaldehyde
was therefore refluxed with KOH and spirit under the same conditions as those
of tissue saponification. The resinous product was extracted from the saponifica-
tion mixture with ether and injected into mice in the usual way. In another
experiment the resinous product before injection into mice was mixed with
cholesterol, which was intended to act as a carrier, in order to imitate rather
more closely the products and conditions after tissue saponification (Experiments
1 and 2). All the tests on resin gave negative results.

Although in the earlier experiments spirit was used for the saponification, a
number of unsaponifiable fractions made from adrenals, cancer liver and non-
cancer liver (see note to experiment 3a for a definition of these terms) were found
to be non-tumour-producing, which suggests that carcinogens or precursors are

DESCRIPTION OF PLATES.

FIG. 1.--Sarcoma in a C57 c mouse at site of injection of commercial cholesterol in lard

(15 per cent). 11th month. Experiment lOd. This tunour is now in its 63rd genera-
ti on. x 55.

FIG. 2.--Same tumour as Fig. 1. x 290.

FIG. 3.-Sarcoma produced by commercial cholesterol in a C57 3 mouse. 20th month.

Experiment lOd. x 55.

FIG. 4.-Same tumour as Fig. 3. x 290.

FIG. 5.-Sarcoma produced by commercial cholesterol in a MNRC c mouse. 23rd month.

Experiment lOd. x 55.

FIG. 6.--Sarcoma produced by cod liver oil in a C57 ? mouse. 17th month. Experiment

10b. x 55.

FIG. 7.-Sarcoma produced by a fraction from lung-kidney-muscle of non-cancerous human

subjects. The fraction had been saponified 3 times, crystallized from MeOH 3 times and
once from acetone. 16th month. Stock c mouse. Exeitiment 3f. X 55.

FIG. 8.-Sarcoma produced by lard in an old C57 i mouse vhich was 16 months at the begin-

ning of the experiment. 6th month. x 55.

124

BRITISH JouRNAL OF CANCER.

Hicge.

VoL IIH, No. 1.

4-

BRITISH JOLRN-L OF CANCER.

l,i'- -tis

oi_    s %

; -e ';

a;''0~\

*I;4
_ N % .

Hgcr.

VoL m, No. 1.

CARCINOGENIC ACTIVITY OF LIPOID SUBSTANXCES

either absent in the reagents or are present only occasionally. If the carcinogen
were derived from the spirit a constant carcinogenic factor would be present.
An alternative explanation would be that, in the experiments just mentioned,
the mice happened to be non-sensitive. The question of sensitivity will be
discussed more fully later on.

3. Fractionation of cholesterol rich (c. 85 per cent) carcinogenic fraction from human

tissues.

Since the carcinogen was concentrated in the c. 85 per cent cholesterol fraction
of the unsaponifiable part of tissue, a number of sub-fractions were prepared and
tested on mice, thus:

(a) Eighty-five per cent cholesterol fraction (fraction A) from pooled
cancer livers was recrystallized from hot MeOH and cooled to room
temperature instead of to - 10?; crv-stals and mother liquors were tested
separately on 20 and 15 C57 mice respectively.  In the 20th month a
single tumour appeared in the "crystals" series (Table I).

Note.-In this paper "cancer liver" indicates the liver of a human
subject who has died of cancer of any part of the body. "N on-cancer
liver" indicates the liver of a human subject who has died of any cause
other than cancer.

(b) Fraction A from mixed cancer livers, partly the same as those
used for the preparation in (a), was crystallized from 80 per cent aqueous
alcohol after adsorption from petrol ether on to a large column of A1203
eluted with petrol ether-benzene-MeOH (80-20-20). The 80 per cent
aqueous alcohol cr-stals (glistening plates m.p. 130-135?) were tested on
10 C57 mice; the mother liquors sufficed for 5 mice. In the 19th month
a sarcoma appeared in the crystal series.

(c) A more detailed fractionation of fraction A from nmixed cancer
livers was carried out as in Flow Sheet I.

The fractions A, B, C, D, E gave no tumours, although of 40 mice
initially, 24 survived 21 months; for survival rate see Table I. The
possible reasons for the absence of tumours are discussed below.

(d) A similar fractionation was made on another batch of cancer livers,
and mice were injected with material from G, C, F, D, E and H. No
tumours arose, although of 50 mice, 48 survived for 12 months, and 32
for 18 months. The test on F was repeated on a further 10 mice in which
one tumour arose after 2 years.

(f) The - 10? fraction from human non-cancer lung-kidney-muscle
(fraction A) had given 3 tumours in 25 mice, and was considered a potent
carcinogen-containing fraction.  It had been in the cold room for over
3 years and was then fractionated according to a simplified scheme (Flow
Sheet 2).

Of 52 mice at the beginning of the tests, 43 survived 12 months and
26, 18 months. Four sarcomas appeared, 3 in fraction G and 1 in C.

(g) The crude unsaponifiable fraction (M) of mixed livers which had
been used in experiment (d) was fractionated and tested at J, K, L, G, A,
C, F, M, I, using in all 110 mice, which lived well; 92 survived 12 months
and 40, 18 months. Two tumours were produced, one by the fraction

125

126                            I. HIEGER

FLW   S   ET I.

human tissue (liver, lung, lkidney muscle)

saponified twice

unsaponifiable fraction ............... .  Stage M

extracted with boiling MeOHL

I

MeOlH insoluble ......................   J

MeOH moderately soluble ................    K
MeOL soluble
cooled to - 10?

and filtered

I

:      crystals ................................   A
~~i       (= c. 85 per cent cholesterol)
mother

liquors .       ............................................  L

resaponified

3rd saponification stage... ..................  B

i crystallized from hot MeOH

I

MeOlI crystals ..........................   G

m.p. 135-7?
MeOH mother

liquors

concentrated

crystals ..   .   ..............................  E
mother liquors .................................   D
insoluble .....................................        I

crystaUized
from acetone

.I

acetone

crystals ............................  C
m.p. 143-4?

acetone

mother liquors     ..........................    F

CARCINOGENIC ACTIVITY OF LIPOID SUBSTANCES

FLOW SHFET 2.

-10? fraction ........................  Stage A

resaponification (3rd)

crystallized from

hot MeOH

crystals ..............................  G
MeOH

mother .......................................  N

liquors

crystallized from

acetone

crystals ..........................C
acetone

mother                 ...                  F
liquors

obtained at the same level as in (d) (i.e. mother liquors of acetone crystals,
(F)) and one by the crystals from which these mother liquors had separated
(G).

(h) Fractionation of 85 per cet choleserol fraction via aeylation (in
collaboration with Dr. C. W. Shoppee).-In two earlier papers (Hieger,
1940, 1946) it was reported that the unsaponifiable fraction of the livers
of Bantu natives contained carcinogen. A large batch of the crude
unsaponifiable matter was worked up to stage A; a part was kept for
injection, and another part was acetylated and crystallized by careful
seeding. The crystalline acetate was hydrolysed and the regenerated
cholesterol tested. 20 mice were employed in each test. In the 12th
month one sarcoma appeared in the A series, and in the 19th month one
sarcoma developed in the cholesterol group.

(i) Cholesterol-rich fraction from cream (cow's milk).-Kennaway (un-
published communication) had found that the unsaponifiable part of
cow's milk produced a sarcoma by injection into C3H mice. A more
concentrated source of the unsaponifiable fraction was obviously milk fat.
3 kg. cream was therefore saponified twice, extracted and crystallized
from MeOH to give stage A fraction which was injected into 20 mice.
Stage L fraction, i.e. mother liquors of A, were also injected into 10 mice.
Two tumours appeared in the crystalline A fraction series in the 13th and
19th months; no tumours were found at the site of injection in the L
series.

(j) Bile and gall bladder as sources of carcinogen.-According to one
hypothesis of human cancer, carcinogens are formed as a result of faulty
steroid metabolism, consequently bile, which is a rich source of steroids,
might well contain the aberrant factor.

127

I. ]IEGER

Forty-five gall bladders from cancerous subjects were used; the bile
was not separated, and some of the bladders were almost empty. Bladders
and bile were saponified and worked up as before and the fractions tested
at C, D, E, F. The experimental mice lived well; after 29 months a
mouse of the F series (Flow Sheet I) developed a sarcoma at the site of
injection.

(k) Liver from a lymphosarcomatous human subject gave a highly
carcinogenic unsaponifiable fraction in some of the earlier experiments
(Hieger, 1940), and the tissues of humans with this type of cancer may
possibly be rich sources of carcinogen Three livers from lymphosarcoma
cases were saponified, fractionated into stages A, J, M, L, and injected
into 6, 5, 11 and 6 mice of crossed C3H x C57 strain, which survived
well, but no tumours developed.

Experiment 4.

The 85 per cent cholesterol fraction (A) of cancer livers which had given 2
sarcomas in 10 stock mice was stored in the cold room for 2- years, then tested
on 10 C57 mice. No tumours arose, although 3 mice survived 24 months.

Experiment 5.

Dr. Mead, of British Drug Houses Ltd., kindly sent us for testing 5 fractions
consisting chiefly of cholesterol obtained from cattle liver in the preparation of
vitamin A concentrates. 119 mice were used in all for these tests but they did
not live well; 60 survived for 12 months and 18 for 18 months; no tumours
resulted.

Experiment 6.-To test if commercial cholesterol could replace the " cholesterol-rich

(c. 85 per cent) fraction."

Mixed human livers, some cancer, some non-cancer, were saponified and
worked up to give the -10? (i.e. 85 per cent cholesterol) fraction (A) and also
the mother liquors of this fraction (L). Commercial cholesterol was then added
to L to give the same concentration of sterol as constituted the unsaponifiable
fraction before the separation of the crystalline part A. Three series of mice
were set up:

(1) injected with -10? fraction (A);

(2)   ,,    ,, mother liquors of -10?, i.e. L;

(3)   ,,    ,, L to which the appropriate amount of cholesterol had

been added.

Of 32 mice originally 28 survived 12 months, and 14, 18 months; no tumours
resulted.

7. Effect of solvent.

Sesame oil is commonly used as a solvent for the injection of compounds
undergoing tests for carcinogenic activity. Gardner (unpublished communica-
tion) reported that he found a single sarcoma in control mice injected with this
solvent. Steiner, Warren Stanger and Bolyard (1947) obtained one sarcoma in

128

CARCINOGENIC ACTVITY OF LIPOID SUBSTANCES

145 mice injected with tricaprylin, and Harris (1941) found that wheat germ oil
gave rise to sarcoma on injection into rats and mice.

Two series of mice were set up; one was injected with sesame oil containing
10 per cent commercial cholesterol, and one with the oil alone as control. One
sarcoma appeared in the first series in the 14th month in a C57 female, and one
sarcoma of fibrous type in the second in the 22nd month in a stock female
(Experiment 7, Table I).

When over 300 control tests had been carried out on lard alone without the
production of a single tumour, a sarcoma arose at the site of injection in a C57
mouse first injected with lard when aLready 16 months old. The latent period
was 6 months, the shortest observed here in all the tests on carcinogens of
biological origin.

Old mice were used in this experiment to see whether the prolonged latent
period for tissue carcinogens was equivalent to the interval required for some
essential preliminary change in the tissue which prepares it for carcinogenesis,
and whether the natural ageing of tissue could in some degree be a substitute for
the latent interval.

Earlier experiments in this Institute had shown that sarcoma may occur in
rats injected with lard alone when strict precautions were taken against con-
tamination by hydrocarbons (Burrows, Hieger and Kennaway, 1932, 1936).

8. Fatty extract of Walker rat tunmour.

Aptekman, King and Lewis (1943) induced sarcoma in rats by injection of
the fatty fraction of Walker rat tumours. Their results were confirmed here by
tests where the tumours were dried by storing in distilled spirit and extracting
with benzene. The crude benzene extract was evaporated, and the residue
without added solvent (lard) injected subcutaneously into 10 wistar rats. A
parallel group of 10 rats were injected subcutaneously with lard alone. After
two years one rat in each series developed a fibro-sarcoma, which grew with
difficulty on transplantation. The tumour in the lard series was more malignant
than the tumour in the Walker extract series. Both tumours arose in the sub-
cutaneous tissue at the same level as the injection site, but on the opposite side
of the body. This effect has been observed several times in experiments where
the injected substance is very fluid, and is probably pressed round the sub-
cutaneous spaces by the movements of the animal

9. MisceUaneous tissue.

The ether extract of minced mammae from lactating mice .(DBA of high
mammary cancer strain) proved carcinogenic, for it gave 1 sarcoma in 10 mice
(Hieger, 1941). An ether extract of the lactating mamma of the rabbit was
evaporated and the oily material injected into 20 mice, but no tumours resulted.
Two sarcomas were obtained by injection of an ether extract of human cancer
liver (Hieger, 1946).

10. Carcinogenic activity of commercial cholesterol.

The carcinogenic activity of commercial cholesterol (about 95-98 per cent
pure sterol) has been briefly described (Hieger, 1947). Tests of the commercial
product dissolved in lard were begun when it was found that the fiactions of

9

129

I. HEIIEGER

"000000  00"000  0020"40-
_  o  o  o  o oo oo_ ......  .....

oo  oooo  ooo

i.   0 0  2  "002C'. . .  0 0 00

00 t qo  to 10  e -  la to  OD 00 e

o

.   ._ . . . . . .   .. . . .

t.~ t,o w  line   n se

t0  0  Ioo O o00 No  0 0 I0'

I~ ~ ~ ~ ~ ~~ - IL  I L ... D

0cis CD  ci  Ca o  o   t0
~~~~~~~~~~~~~

02..  .0  .  .   0 *  .  *  .  .  .  *  *  . ,.

.6 =   o   0t000   000

o  *  ....  -  -   .  . .  .

V O            ::  ?:::

0 ~ ~ ~

-.4 ~ ~ i~

-- *.  . 1 . ' .1 *'"' * . * *

~nm  . ?  -4 Q   X   :

0

X2
C)

0

a)

.0
:4

0

0

0

0

5-

co
0.

0                    0

0                    0

0                     0

C)

@2
._

0

:4

0
0

0

0

CD
0

cis

@2

0

0
0

?t

r._

0 _
_ m

_4

>0

00

-0

-. . *0 *-*

. . .. .m . .~e

-    - . . -. -. -.

40400 4 00004Q 0410

o   .  .C  .  .  -

_i m.?  .I" .ou  .?  ._e

00

"4f  "4  14 0

o -
0 0. -0

* t

2 0. 02
0-

40

Q   o  O0 0

.  to   :

V      V

0~~~~~~

o                   CD

0~~~~~

o D               4

~~~ o

@2             @ 44 00

O 0        o~~0

00~~~~~~~~~ 0

130

E-

: 0

a

.0
0
0

0

I

04
04

to
0Z4

,--eO
-O40
"40

00
0 0

_ _4
*

00 -
0 0
04 _-
C) t,
tll ill

.4 4

a0
qD

.9

0

-"P

02
.e
0
o~-
-"

0

Ib

1,

o~.0

:

n~

.Ia
,%a

o - 0

X 5 V:

?o

r._

cc

_0
0

..4-
,--4 4
r. 0

-;"

4 50

tt

Ii

o IC
0 0

,Q -

C0

la Cc;
0

0
0

E .2 ;d

$.'A

:
02

ft

T!

o ?' 0 "

0, 0 o -

0; o.

C    0D
0

0 ..o4

0?     0

0

0 0

CARCINOGENIC ACTIVITY OF LIPOID SUBSTANCES

oo0  o0000 0 0 0

00 .Z .

O o e* e
0, loC

co   _-4 Cq

* cc C4 r-    a

0

~ Go 0 0 -

ma go0c G
V-    P-

@1Q ) 0 00
p. P- -4 -

? -  * -

8 0 oooo)
Ar    _ _ _- _- -

0
C0

Ca        C o

0   ~  4m
,m4

0   0  0

0" 0. ,4

t-             t

ic: :: .0 ::. :i

o)      >b

2      .a      0
o  :::  0::._

P4:    P4      O

4--
P-   P-     0 0 C)

o   o

*   .    .  -

I  I    C

-  -   -  .- I.

. 11.

,m -4 -e,-41  4.0l,-  ,, -.
C- -Q     - *o

?  . . . . . . o *

oooo    oooo

_ _0 _ _ _ _ __

0  .   .  * *E  *-0

000     0000EXV52V

c .. .  .   .

~o o~

C ~o o    .0

0 C
rs ?

o~~~~~~~

0 ~~~~~~~00= 40

0

0      4~~~~~~~~C  00      co

-I- ?. ~   o     '"1

0           o~0-~ oo_ 0  o5? r~

-  .    _

0  0

">--          0 0  0 0

~2~4

134

U0

+

0

U-

o.

00 o#

*e 0
0

0 o

?O ?? ??- o  ? -

~00000-4t   -

-,0

.   .0* *

cq 0 0   O X  *

lo 0 : : :   :  :

ooooo 0o ot

_- 4   -_  _4 _-   _-  _

0000  o lto  0  0  C

*      * . * . .

P-4 -44--  -4  0-4P  -4 -  *- P-

00000  ~ ~0 0 0 0 0

.  . * . . *   .m   .

o      FO

0.  05

60 .     0=   0   0      0D~

0 4 04,4 :4

*0=4    0.

0-    0

0 : 4

ew

0
Cq

P-

Z bO 0             0 t
X Z P- -- P- P- 0 6   e=  C

-

H1 4     4 >  >  "    0      0..4         >o

1-             "m     -      -

0        0

d

8
E-4

131

01

@1
uS

I      A

4

Eq

a
m

.

I. HIEGER

01-

o o   - o.

? ,.   o
CD

ci  *  0
si  .

.   0

.0 ~ ~  .

o  - e
s   *

,.? - 01 >

_* _ *
N Cl

C^..
ci d

C   C

cc"

2

Ca 00   0

O

C.  - 2 f-.

0

Ca
0

o 0

ce

--      0

.0~~~

-o
0 o

G ~t

0

CD
.-

-I
I

-C-O O o, O _ IC   o -

- 0. .  .0 .o .0 u.  O   .,. .,. .  *  . . . . . -

?1.
O * * * * * * **  *  O

?  *  . o  .  .  *  . .  * . ....  *o  .  .  . . ?
0 * -C O   -  O*

.  .       .......o *

0:: 'O : - 0  : :  e:  :::  *  *  -

r .  .              .

a  eao e      <*tov

. ** .....**.*.

0

0

r.

0

Cd

c
tD

a

.r..

0

-

-

C)
0

5
0
0

le
0

132

-o

I-I

I

E--

o

0

04
0

.0

-00

0

Ca D

0

0

CB

0.,

0

> O

It 0
O O

.0 _

0.0

m cc
O

cc   I

O0

O > O -b

0      C 0

0 0 ~0

0. 0

0

IC

o; o

0     0

t
C   o

X .0  P c0

.CP; 0
*

0 ,o

0-   '?

E-  r.4

~o

~D

CARCINOGENIC ACTIVITY OF LIPOID SUBSTANCES

tissues which were richest in cholesterol were also the most potent carcinogenically.
Up to the present 25 sarcomas have been obtained in 436 mice by commercial
cholesterol or some fraction of it, in solution in lard or in other solvents (Table
I (lOd), and Table 11).

TABix 11.-Sarcoma Production by Commercial Choesterol.

Number of mice.

*ateraL                              A        Sarcom*.

L  12 mnh

1. Commercial cholterol .  .      .   .   .    .      213      77  .  11
2.     ,              ses ame oil  .  .   .    .   .   20      16  .   1
3.            -     + cod liver oil  .  .  .   .   .  100      71  .   7
4.             ,    (after   xing with KOH in spirt (or

alcohol)).  .   .   .   .   .   40      35   .   2
5.                  crystallized from acetone  .  .  .  63     41  .   4

Total  .  .   .    .   .   .   .    .   .   .  436     240  .   25

* Injections of 15 per cent solutions in lard except for No. 2.

10a. Effect on cholesterol of refluxing with KOH and alcohol.

The possibility that carcinogens are formed from cholesterol during saponifica-
tion was tested as follows: 10 g. of commercial cholesterol (i.e. about the amount
present in a human liver) was refluxed for 2j hours with 400 g. KOH, 1 1. distilled
alcohol or spirit and enough water to imitate the conditions in the saponification
of a liver; the mixture was extracted with ether and injected into mice as before.
A preliminary statement (Hieger, 1947) was made, but further series of mice
have been used (10a in Table I).

Four groups of mice were set up:

a. 10 C57 mice injected with cholesterol saponified in distilled spirit.
b. 10 stock     ,,            ,, ,       ,    ,

c. 10 C57        ,,           ,,          ,,        ,,     alcohol.
d. 10 stock     ,,            ,,    ,

In the 14th month in group b 2 mice developed sarcomas, and pre-neoplastic
changes were seen at the site of injection in 4 other mice.

Thus in group b, 6 of 10 mice had either developed sarcoma or might have
done so if they had lived longer. The two sarcomatous mice and two of the
incipiently sarcomatous mice were from the same box of 5 mice.

One cannot say why only group b responded so readily, but (1) in a fair
number of experiments the saponification of tissue has been done in spirit but
the products have produced no tumours, showing that spirit is not essential for
carcinogenesis by the unsaponifiable product; (2) the absorption spectra of the
two final products (cholesterol saponified in spirit and in alcohol) are closely
similar; and (3) while the substance was strongly carcinogenic in group b (stock
mice), the same preparation was inactive in group a (C57). Consequently it
must be supposed that the b group of mice were either

(1) Peculiar genetically; (2) peculiar congenitally; (3) had become
infected or (4) had become contaminated with carcinogen.
(4) is, on the whole, not very probable, because -

The 1946 paper reported that in tests on 18 subfractions of the unsaponifiable
fraction of human tissue, partly from cancerous, partly from non-cancerous

133

I. IEGER

subjects, of the total yield of 8 sarcomas, 7 were produced by fraction A made
from 3 different sources and 1 from an M fraction (Flow Sheet 1). The concen-
tration of 7/8ths of the sarcomas in one particular fraction strongly suggests
that the fraction itself was responsible and not a chance contamination, for it
would be highly improbable that contamination would occur only in the boxes,
holding 5 mice each, containing those treated with the A fraction.

The data in Table mI are the results when a fraction was divided into 3 or
more sub-fractions. The concentration of tumour production in a few fractions,
particularly F, G and A, supports the conclusion in the foregoing paragraph,
namely, that contamination will not explain satisfactorily the incidence of
sarcomas. Contamination only in those boxes where these particular fractions
were used would be very unlikely, since each box contains 5 mice initially and
the experiments on these fractions were started over a period of 4 years, during
which time the boxes were changed many times (Table I).

TABrLa m.-Showing Trend of Concentration of Carcinogenic Factor

During Fractionation.

Number of

Ycm      sampes deaived  Nmber of   S urvivm      Number of     Number of

(see riow  from diferit  mice at     at 12     .  sacoms       sampnes which
Sheet 1).  batches of   start.       mt                        gave podve

timue.                     ?  ---    ~    ~~~~~~~ resinir.

A     .     11     .    175    .    110    .   10      6     .   5
M     .      4     .     52    .    41     .    1      2     .   1
J     .     4      .    40     .    25     .    0      0     .   0
L     .      8     .    141    .    101    .   0       0     .   0
G     .     3      .    30     .    26     .    4     13     .   2
C     .     5      .    57     .    52     .    1      2     .    1
F     .     4      .    45     .    37    .    3       7    .    3
I     .     2      .     10    .     9     .   0       0     .   0
D     .     3      .    25     .    24     .   0       0     .   0
E     .      1     .    10     .    10     .   0       0     .   0
K     .     2      .    20     .    18    .    0       0    .    0

lOb. Effect of addition of oils on carcinogenic potency of commercial cholesterol.

The activity of commercial cholesterol suggested several possibilities; for
example:

(a) That cholesterol is indeed a feeble carcinogen.

(b) That commercial cholesterol as well as the cholesterol-rich fraction
obtained from a number of sources contains a small amount of a potent
carcinogen.

(c) That a combination of cholesterol, as carrier, and an active agent
are essential for carcinogenesis.

(d) That all the preparations of cholesterol used are contaminated with
atmospheric carcinogen (i.e. benzpyrene from soot).

(e) That carcnogens of biological origin such as lipids and particularly
preparations rich in cholesterol evoke neoplasia, but only in the most
susceptible members of groulps of experimental animals.

Tests of hypotheses (a) and (b) are to be described more fiully in a forth-
coming paper. Highly purified cholesterol, prepared by Dr. C. W. Shoppee,
using bromination, crystallization of acetate and chromatography, has been

134

CARCINOGENIC ACTLIVITY OF LIPOID SUBSTANCES

injected into 25 mice; the experiment had been in progress for 22 months
without result as yet. (b) is being tested by concentrating the impuritities in
commercial cholesterol 3 times and in a second experiment 10 times, and inject-
ing the products. These experiments have been in progress for 11 months
without result as yet. (c) was investigated by mixing oils containing reactive
double bonds (i.e. unsaturated) with commercial cholesterol to see if the activity
could thereby be increased (Table I, experiment lOb).

Cod liver oil certainly increases the potency of cholesterol dissolved in lard,
but since at least one sarcoma has now been obtained with cod liver oil alone
(Table I, 10b, series x), it is uncertain whether the oil is a co-carcinogen or a
carcinogen.

10c. Effect of simple crystallization on the carcinogenic potency of commercial

cholesterol.

The batch of cholesterol used in the preceding experiment was crystallized
from acetone to give 3 fractions:

(1) Lea  soluble.

(2) Moderately soluble.

(3) Mother liquors, which is obviously different from (1) and (2), for
(3) is softer, yellower, more soluble in lard and smells of candle wax.

The three fractions were injected as usual into 20, 21 and 22 mice respectively;
3 sarcomas appeared in the fraction 1 (least soluble) series and 1 in fraction 2
series in the 14th, 18th, 18th and 15th months.

10d. Sarcoma production by commecial cholesterol.

Table II shows the total of sarcomas produced by commercial cholesterol.
lOe. Compounds rdated to cholesterol.

Compounds related to cholesterol and particularly those which might be
present in samples of the impure sterol were also tested, namely, isocholesterol,
isolumisterol, cholesterylene (2 different preparations and also the mixture of
compounds present in the mother liquors after crystallizing out the choles-
terylene), ergosterol, cholestene, 7-dehydrocholesterol (2 different preparations,
one a commercial product containing 60 per cent of the pure dehydrocholesterol),
a mixture of epi- and allo-cholesterol, vitamin D3, and 7-ketocholesterol. Except
for those treated with 7-ketocholesterol all the mice have now died without
tumours being produced; 90 survived 12 months out of 100 initially, and 56
survived 18 months. The test on 7-ketocholesterol has now been in progress for
17 months, without result.

11. Pre-neoplastic changes at the site of injection of lipid.

Since the detection of only a few tumours could be important in deciding the
technique of further fractionation, it was essential not to miss any tumours not
visible macroscopically. The remaining mice (now 700) are examined post
mortem by sections through the fat vesicle, so that any pre-neoplastic changes
might be detected. In 15 of the 700, changes were seen suggestive of pre-
sarcomatous change at the site of injection.

135

136                                I. HIIEGER
12. Steiner's investigations.

In a recent elaborate report Steiner, Warren Stanger and Bolyard (1947)
give further data on sarcoma production by unsaponifiable fractions in three
strains of mice, Albino, A, and C57 (Table IV, A):

TABLEa IV, A.-Experiment of Steiner, Warren Stanger and Bolyard (1947):

Sarcoma Production by Unsaponifiable Fraction of Tissu4s.

Experi-           iaumber                             ce at      Mice at   Sarcomas.

ment.     Unsaponifble fraction of-   ofeas       stat      12 montho cases

ment.                                                start.   12 months.

(pooled).

Cancer cases:

1   .     Human kidney     .   .    .    61   .      50    .    39    .    0
2   .        ,,  spleen    .    .   .    54    .     50    .    47    .     5
3   .        ,,  colon     .   .    .    54    .     49    .    45    .    0
4   .        ,,  heart     .   .    .    63    .     43    .    19    .     1
5   .        ,.  liver     .    .   .    18    .    101    .    45    .    17

Non-Cancer cases:

6   .      lHuman kidney   .   .    .    58    .     50    .    44    .     1
7       .       ,,  spleen  .  .    .   51    .      50    .    40    .    0
8   .        ,,  colon     .    .   .    51    .     47    .    45    .    0
9   .        ,,  heart     .   .    .    35    .     31    .     7    .     0
10   .        ,,  liver     .   .    .    15   .     120    .    39    .   44
11   .  Human liver (stillbor infants)  .  40  .      39    .    34         3
12   .  Pig liver  .   .   .    .    .         .     115    .    82    .    4
13   .  Beef liver .   .    .. .        .            115    .    69    .    0
14   .  Pig heart    .      .   ..       -     .      30    .     9    .    0
15   .  Tricaprylin controls  .  .   .         .     154    .   116    .    1

Total       1044    .   680    .   76

The yield of local sarcomas at the site of injection (7-3 per cent) is of the same
order as that obtained in this Institute, namely 4-7 per cent (63 sarcomas in
1298 mice, of which 936 lived 12 months). These 1298 mice are all those injected
with unsaponifiable material of animal origin, including commercial cholesterol.

In earlier papers, Steiner (1942, 1943) gives results which, taken in conjunction
with the data from his recent paper, can be expressed thus (Table IV, B):

TABLE IV, B.-Experimnens of Steiner, Warren Stanger and Bolyard (1947):

Sarcoma Production: Effect of Pooling of Uneaponifiable Fractions.

Materia                         Ratio: Sarcoma mice total mice used.
1. 8 cancer livers pooled  .  .    .   .    .    .    .   12/56 = 21?o

2. 7 non-cancer livers pooled  .   .   .    .    .    .    5/63 =  8?o
3.15      I's,                .9.       .   .    .    .   44/120 =370to0mean21 ?
4. 18 cancer livers pooled  .  .   .   .    .    .    .   17/101 = 17 o%

5. 37   ,,     tested separately (8 in 37 were active) .   .   12/456 =  4?o
6. 30 non-cancer livers tested separately (6 in 30 were active)  .  10 '440 =  2 3?0o

Thus the effect of testing a number of livers (67) separately was to reduce the
sarcoma production by a factor of 9, i.e. 9 times less than the potency of 48
livers pooled, which is of the order of expectation if it be noted that Steiner (1942)
found 1 in 5 of his livers (cancerous and non-cancerous) to contain the active
factor, and if it be assumed that pooling the livers had the effect of distributing
the agent from the active livers among the inactive ones. Furthermore, it may
be noted that from Steiner's (1942) experiments 1 and 2 one would conclude that

CARCINOGENIC ACTIVITY OF LIPOID SUBSTANCES

cancer livers are of the order of 21 times as potent as non-cancer livers, but from
experiments 3 and 4 it would appear that the ratio of potencies is reversed,
leading to an overall discrepancy as high as 5 to 1. The writer would point out
that three experiments (Nos. lOa and 3f, Table I and Experiment 4) carried out
here suggest that unexplained differences in the susceptibility of the mice to
biological carcinogens can completely alter conclusions as to the carcinogenicity
or otherwise of a substance undergoing test.

TABLE V.-Latent Period* (Month) of Sarconums Produced in Mice

by Injectiow of Lipoid Sub8tances.

Latent        Hieger (1940).   Hieger (1946).    eded     the
period          Number of        Number of      pesent paper.

(months).        tumou     l      tumou           Nmr ofs.

tumoum

6       .       -        .      -         .        1
7       .       -               -

8       .       -        .      -         .        1

9
10
11
12
13
14
15
16
17
18
19
20
21
22
23
24
25
26
27
28
29

1
2
2
1
1
1
2
1
2
1
2

1
1

1
1
1
I

I

1
2
2
6
1
4
3
3
3
3
3
4
3
2
1

1

Total

Total
?           1

?           1

?           1
?           3
. 4
?          3

. ?

?          4
?           5
?          4
?          4
?           6
?          5
?          5
?           5
?           6
?          3
?           1

?           1
?         69

* "Latent period" is taken as the interval between the start of the experi-
ment and the grafting of the induced tumour. The actual initiation time for
the tumour is, of course, shorter. The interval between detection and grafting
is seldom more than a few weeks. Once the primary tumour is well started it
grows with great speed.

Note. The figures in the last column (the overall latent period) show a
periodicity remarkable in such data. It is possible that at the large number
of mice involved, nearly 2000, statistical laws begin to operate.

DISCUSSION AND CONCLUSIONS.

The experimental data given here and in earlier papers from this Institute,
based on 69 sarcomas produced in a total of 1966 animals, present a number of
difficult problems.

No completely satisfactory explanation of the following experimental findings
is as yet available:

(1) Cod liver oil, lard and sesame oil produced at least 1 sarcoma each
in 25, 350 and 20 mice respectively. Other work has shown that some
fats can bring about sarcoma development in sensitive mice or rats.

137

I. HIEGER

(2) Commercial cholesterol in lard gave sarcomas in about 5 per cent
of 213 mice (Table II).

(3) The mixture formed when cholesterol is refluxed with KOH and
distilled spirit gave no tumnours when injected into 10 C57 mice, but in
10 stock mice there arose 2 sarcomas, and in 4 other mice of the same
series changes were detected, post-mortem, suggestive of pre-neoplasia, at
the site of injection. A similar preparation where cholesterol was re-
fluxed with KOH and alcohol gave no tumours when injected into 10
stock and into 10 C57 mice.

(4) Fractionation of an active fraction from tissues by simple crystal-
lization does not always lead to any concentration of the active factor.

(5) Although several experiments showed that the addition of cod
liver oil to commercial cholesterol increased its potency, yet in another
experiment the crystallization of the commercial sterol from acetone,
which removed some waxy constituents, also increased the potency.

The writer has attempted to reconcile these apparently contradictory findings
by putting forward the following suggestions: Firstly, that the operation of little
understood factors covered by the expression "individual variations of suscepti-
bility of the experimental animals " is very important in determining the carcino-
genic potency of the lipids, and secondly, that lipids and particularly sterols or
closely associated compounds are slow-acting carcinogens. It is clear that large
series of mice are required in this type of experiment where the agent is of low
potency, and where, therefore, the presence or absence of especially sensitive
individuals may well be the principal factor in the assessment of the potency of a
test preparation, since in small series of mice the sensitive members may die first,
leaving those which are comparatively insensitive.

The possibility is not quite excluded that lipids are often contaminated by
traces of benzpyrene from air-borne soot.

Thirdly, the existence of about 300 synthetic carcinogens (hydrocarbons, azo
compounds, stilbenes, etc.) suggests that carcinogenic potentiality is probably
more widely distributed among chemical compounds than was at first anticipated,
and thus there is no good reason for excluding compounds of the lipid group.

Fourthly, some of the experiments described here suggest that the carcino-
genicity of lipid substances may either be enhanced or be inhibited by other
lipids.

Fifthly, should the thesis proposed here, namely, that lipids are slow-acting
carcinogens, be conclusively proved, then, "spontaneous" cancer or at least
some forms of the disease could be considered as "caused" by body lipids exist-
ing locally in some peculiar chemical or spatial or temporal conditions.

The production of sarcoma by commercial cholesterol obviously casts some
doubt on the conclusions which have been drawn from all previous experimental
work where cholesterol was involved, and where almost all investigations have
taken for granted that cholesterol is non-carcinogenic. The weakly carcinogenic
potency of a number of other lipid substances and the unexplained variations of
sensitivity of the animals used for testing underline the need for much further
study in this field. The experiments described here suggest the question, Why
has it required 20 years, that is, since the discovery of the carcinogenic hydro-
carbons in 1929, to show that commercial cholesterol is carcinogenic ? The
answer is obvious; investigators in chemical carcinogenesis have become accus-

138

CARCINOGENIC ACTIVITY OF LIPOID SUBSTANCES             139

tomed to the use of powerful, quick-acting carcinogens, and as a result
tumour induction by slow-acting carcinogenic substances has been dismissed as

non-specific."

Control experiments have, in the past, not been done on an adequate scale,
and moreover, commercial cholesterol, having been unconsciously equated with
the cholesterol in the body, leads to the inference that cholesterol is not a
carcinogen since it is present universally. It might be permitted to note, how-
ever, that inferences cannot always satisfactorily substitute experiments.

SUMMARY.

1. A total of 69 sarcomas has been obtained at the site of injection of lipoid
substances in approximately 2000 mice of C57 and MRC strains and commercial
mixed stock mice. The average latent period was about 18 months.

2. 63 tumours were produced by unsaponifiable fractions derived from the
tissues of human subjects (cancerous and non-cancerous) and from cattle; of
the remaining 6 tumours, 5 were produced by non-saponified fat from animal
sources and 1 from a plant source.

3. Commercial cholesterol gave 25 sarcomas in 436 mice.

4. No evidence was obtained to suggest that the carcinogenic substances in
the unsaponifiable fraction is confined to the tissues of human subjects who have
died of cancer.

5. The carcinogenic factor has not yet been isolated in a chemically pure
state. Fractionation by simple crystallization led in one experiment to an
inexplicable loss of potency.

6. The widespread occurrence of a carcinogenic factor in unsaponifiable
fractions from many biological sources suggests that it is either cholesterol itself,
or a combination of cholesterol and a small proportion of a frequently occurring
co-carcinogen.

7. In such experiments the possibility of contamination with benzpyrene
from air-borne soot must be borne in mind.

8. The variations of susceptibility in the experimental mice constitutes a
most important factor in determining the potency of carcinogens of biological
origin.

This investigation has been supported by grants to The Royal Cancer
Hospital, from the British Empire Cancer Campaign, the Jane Coffin Childs
Memorial Fund for Medical Research, the Anna Fuller Fund and the U.S.
Public Health Service.

REFERENCES.

A    xN, P. M., KiG, H. D., XD LEwIS, M. R.--(1943) Cancer Res., 3, 856.

BuRRows, H., HiFGER, I., AND KKNNAWAY, E. L.---(1932) Amer. J. Cancer, 16, 57.-

(1936) J. Path. Bact., 419, 43.

HARRIS, P. N.--(1941) Cancer Res., 1, 751.

HIEGER, I.--(1940) Amer. J. Cancer, 39, 496.-(1941) Science, 93, 262.-(1946) Cancer

Res., 6, 657.--(1947) Nature, 160, 270.

KTIxENXBEgRG, A. E., NEUFACH, S. A., A  SHnD RD, L. M.--(1940) Amer. J. Cancer,

39, 463.

STmNER, P. E.-(1942) Cancer Res., 2, 425.-(1943) Ibid, 3, 385.

Idem, WAMM4 STANGER, D., AD  BOLYARD, H. N.--(1947) Ibid., 7, 273.

				


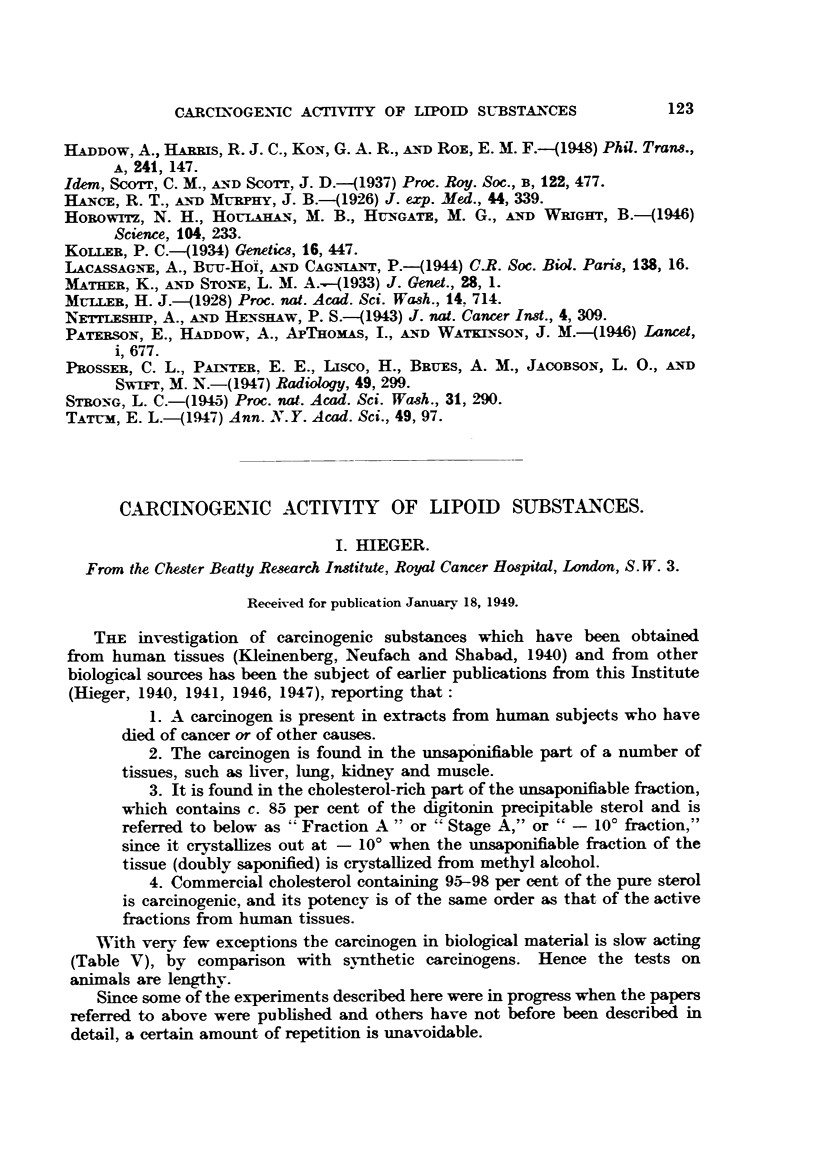

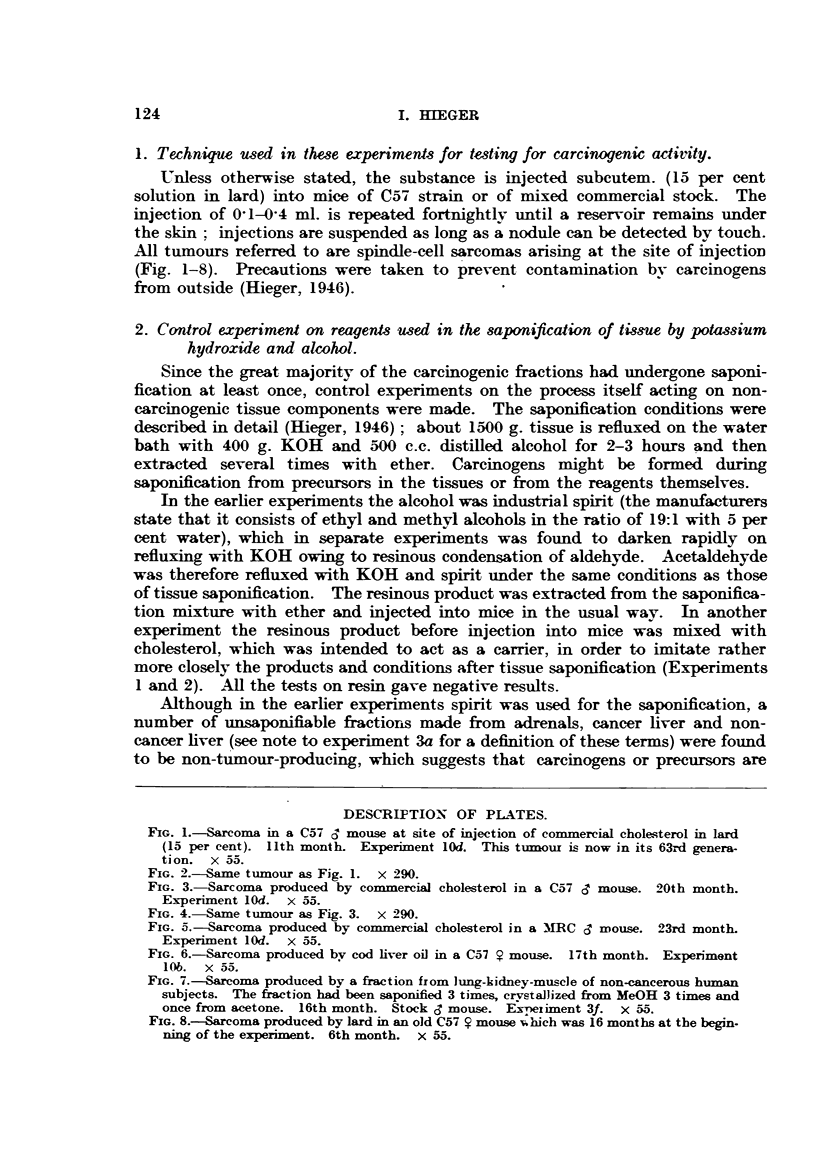

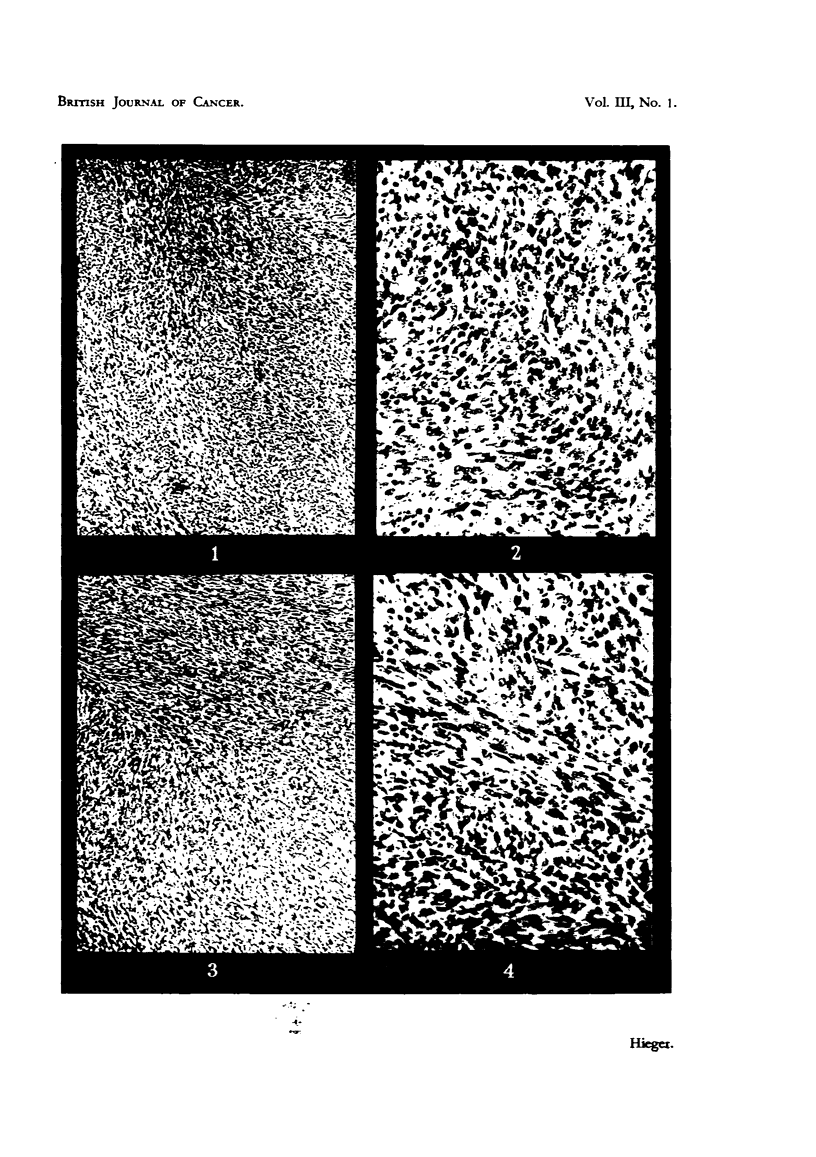

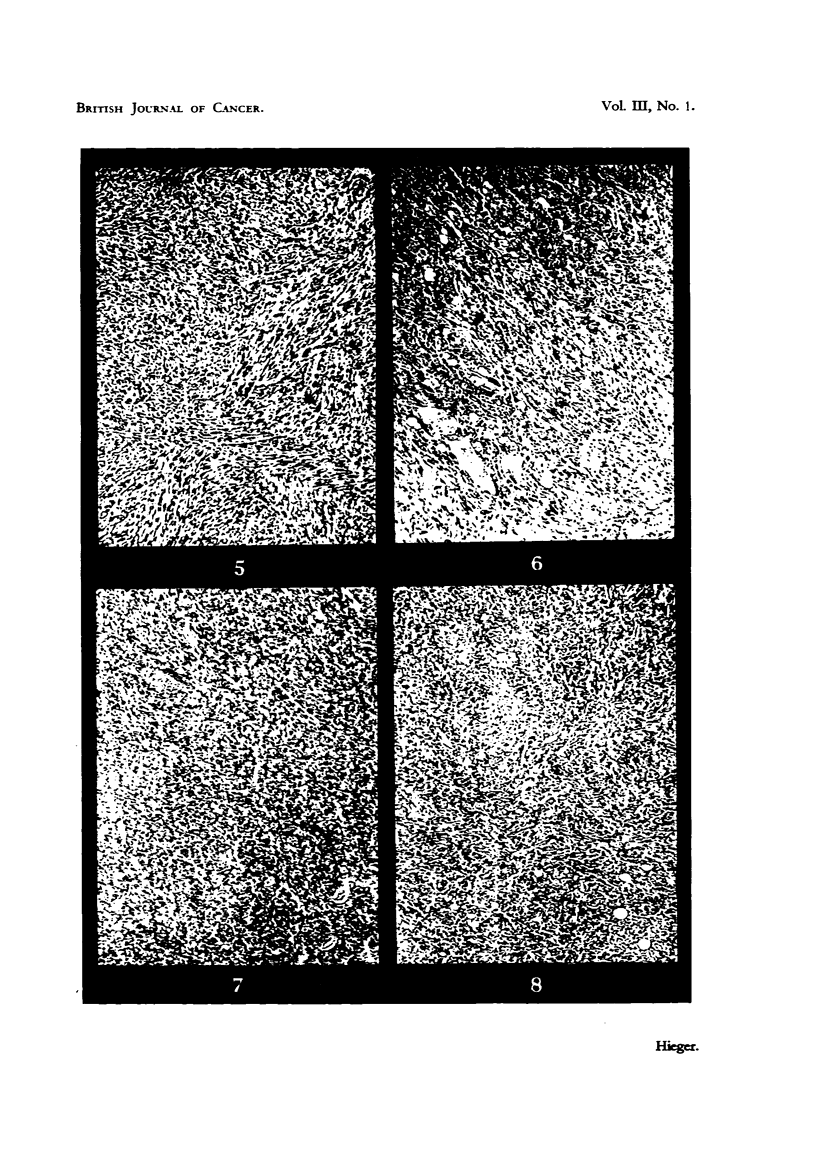

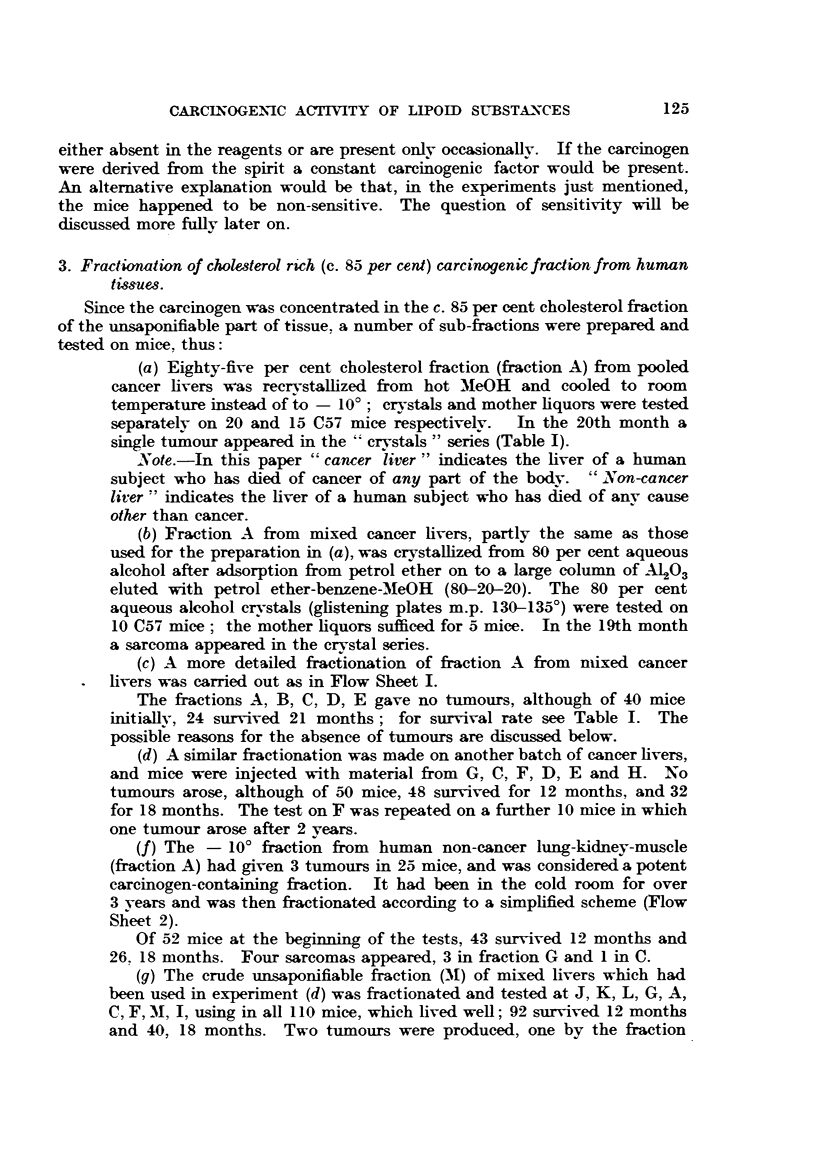

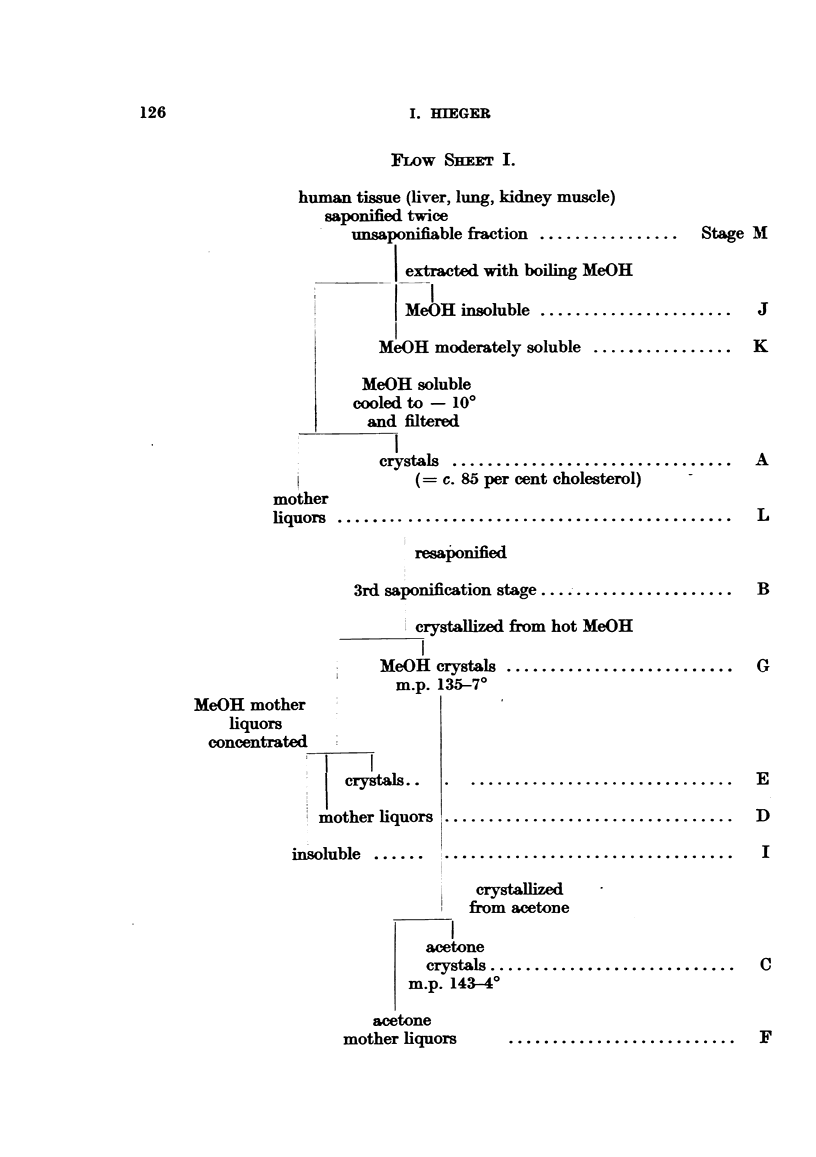

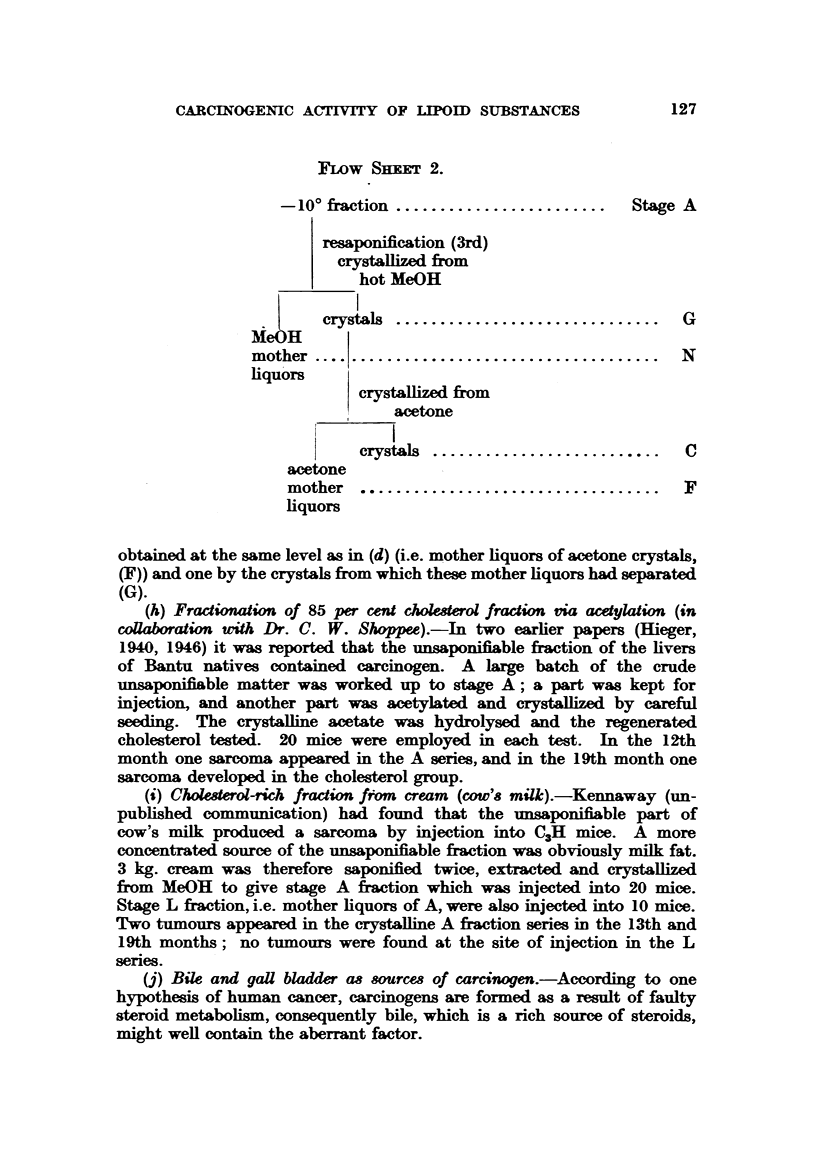

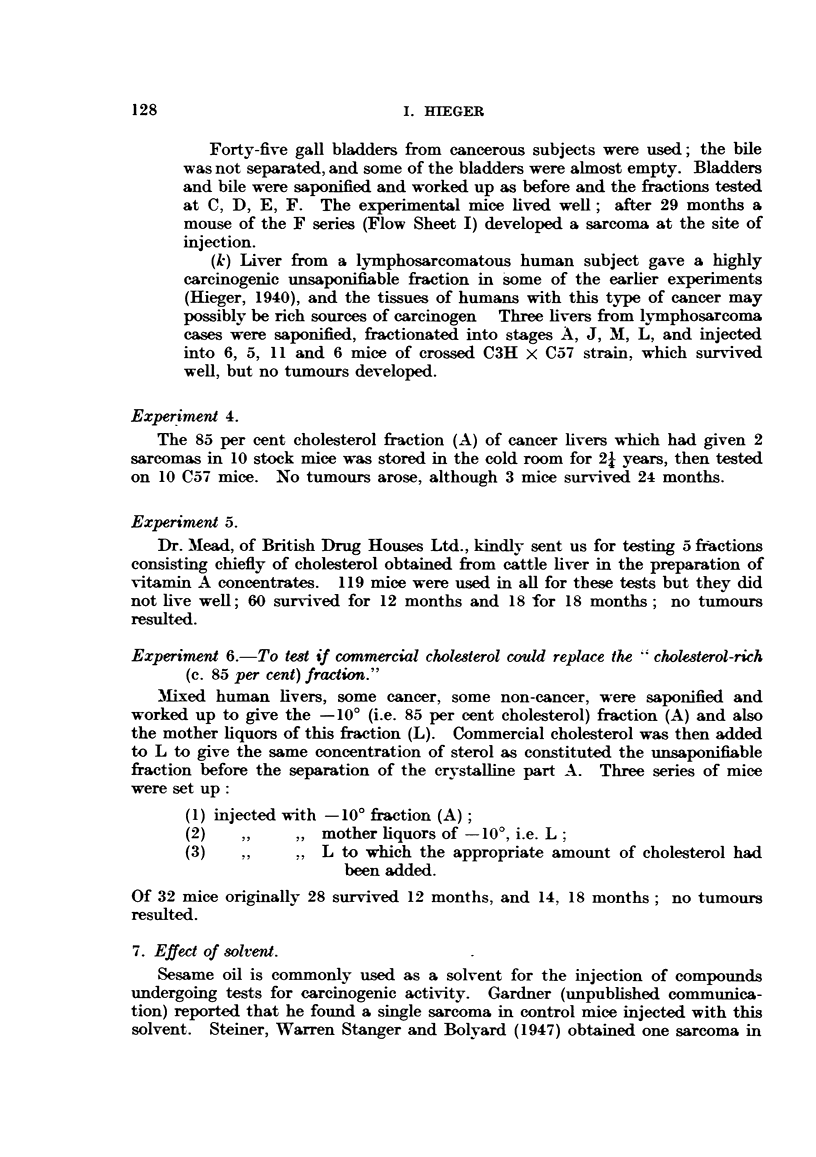

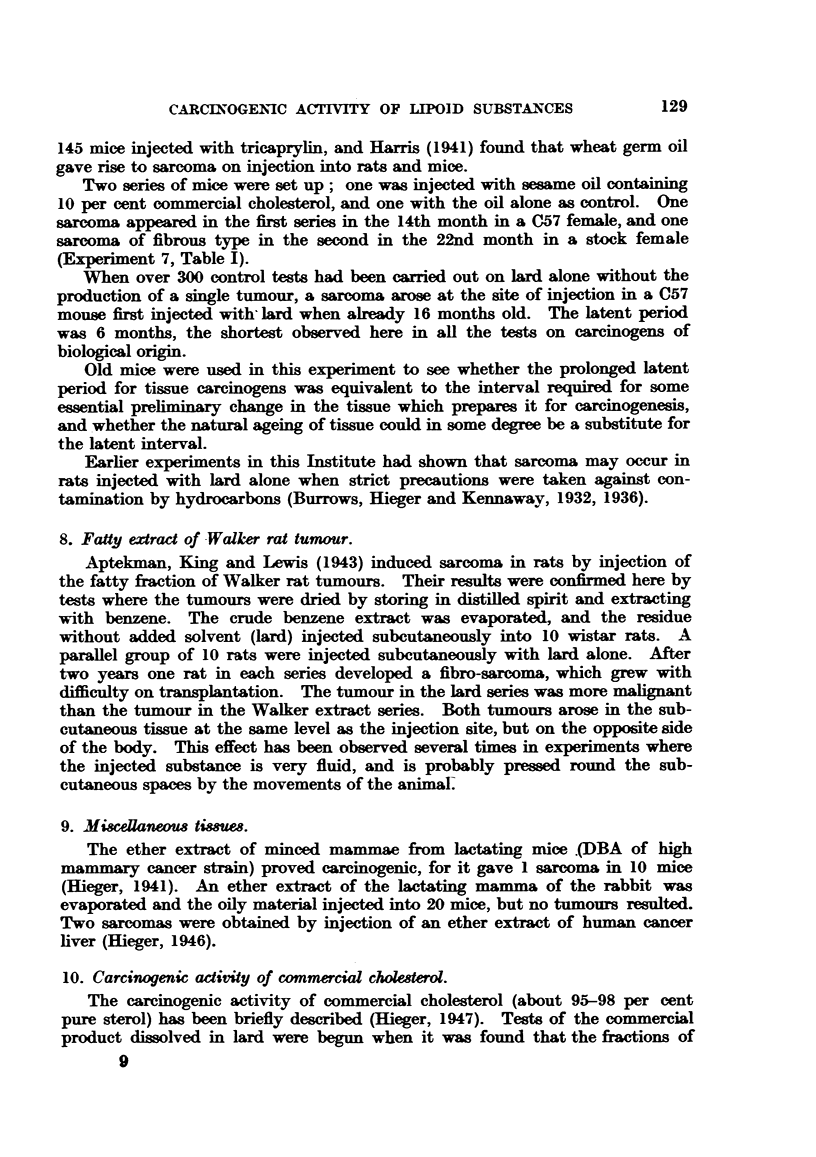

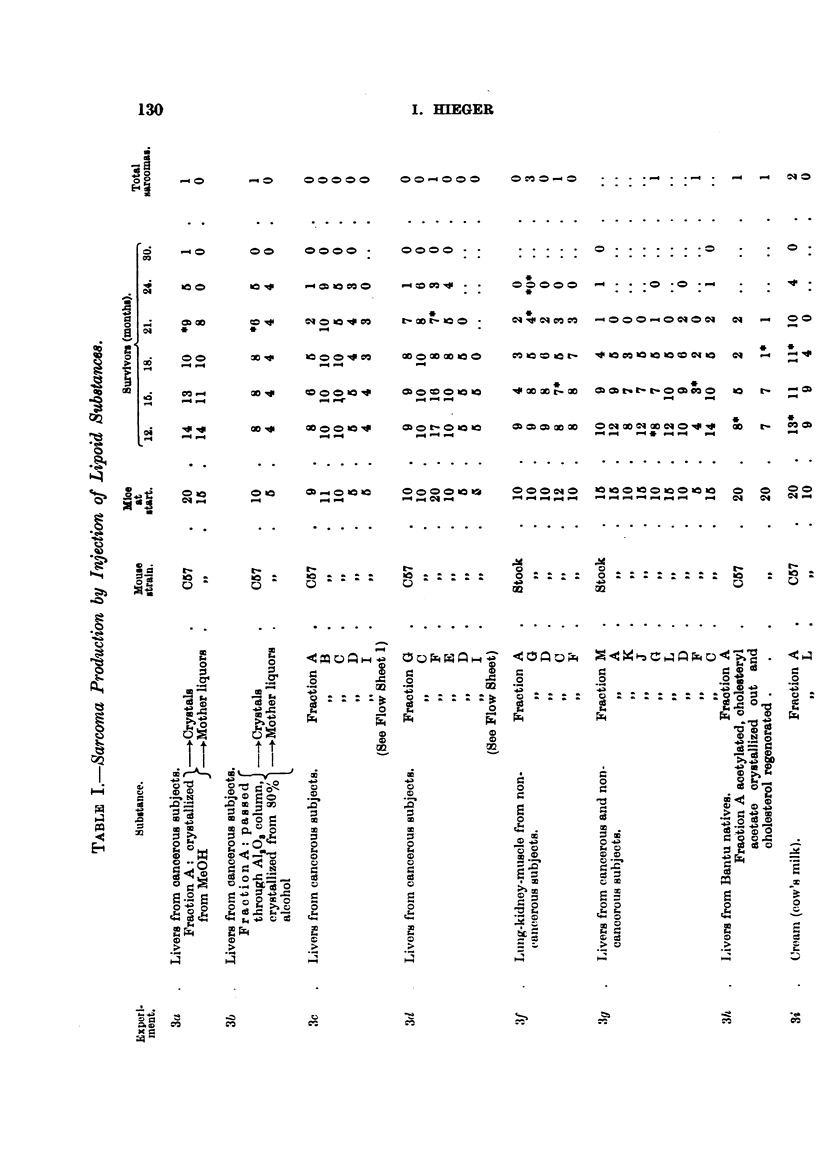

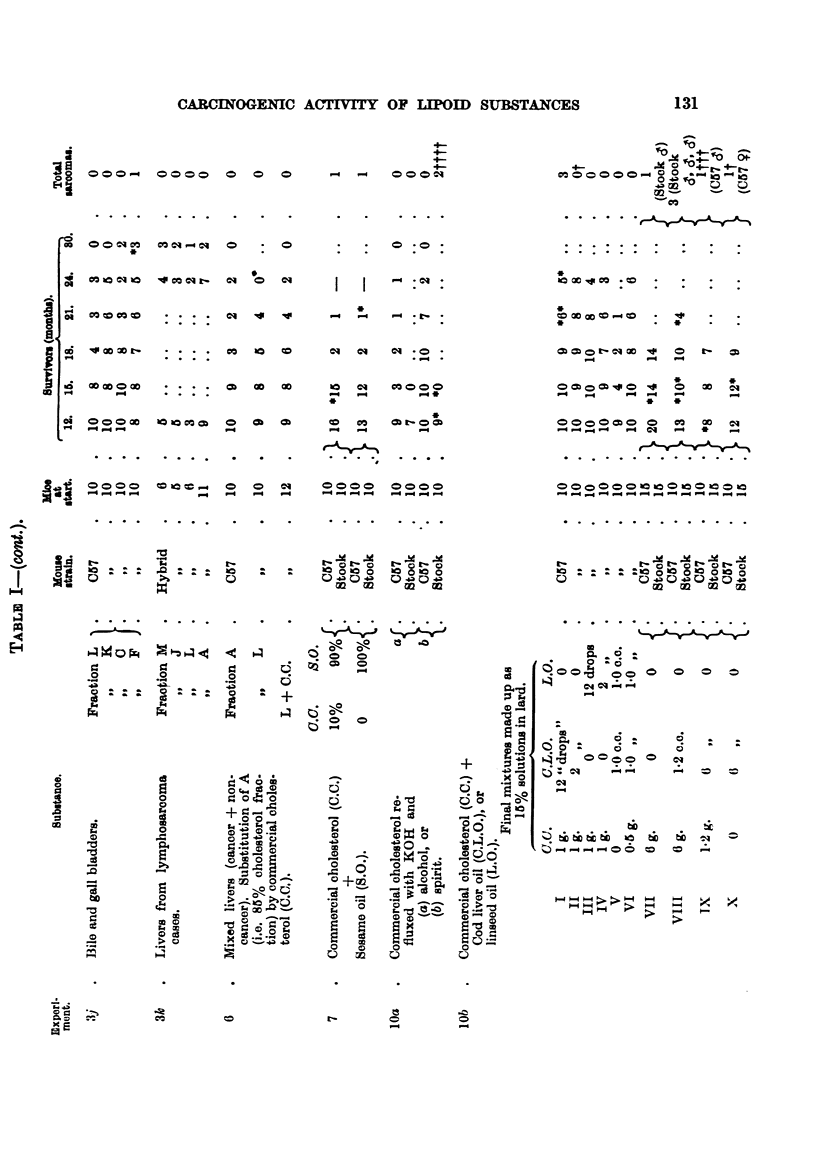

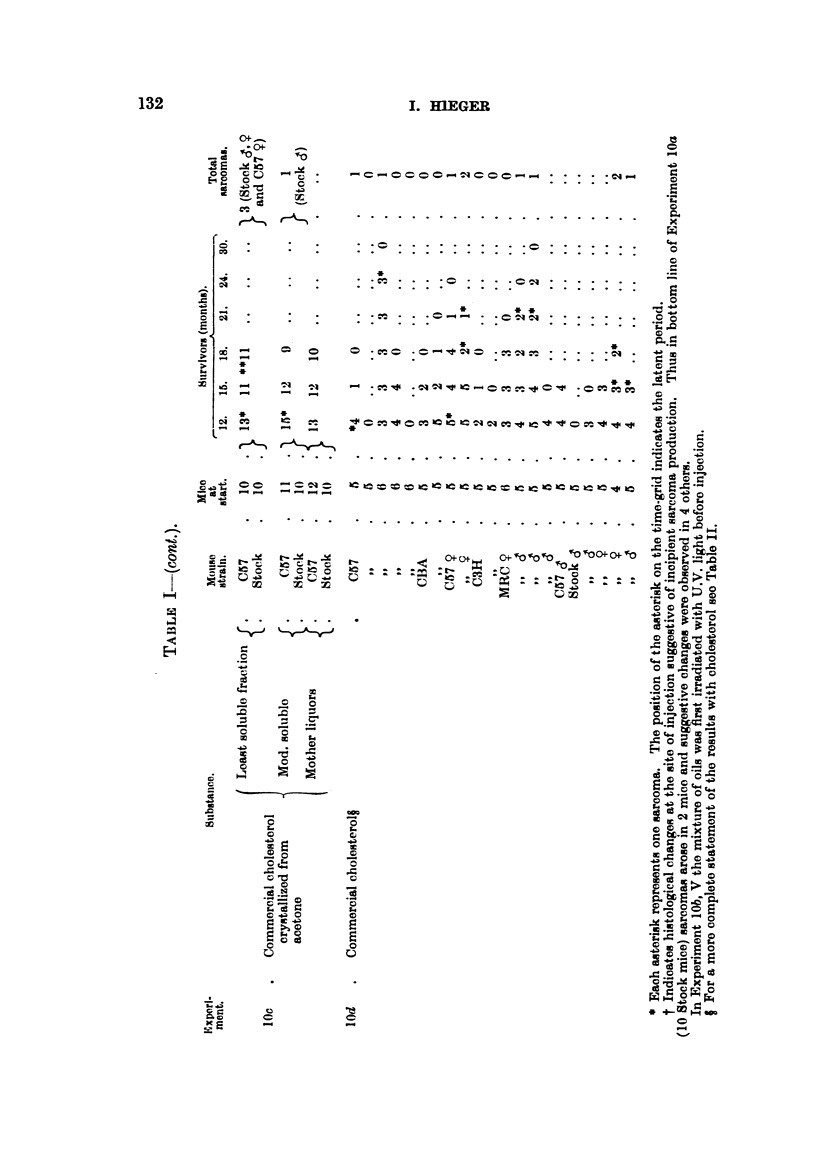

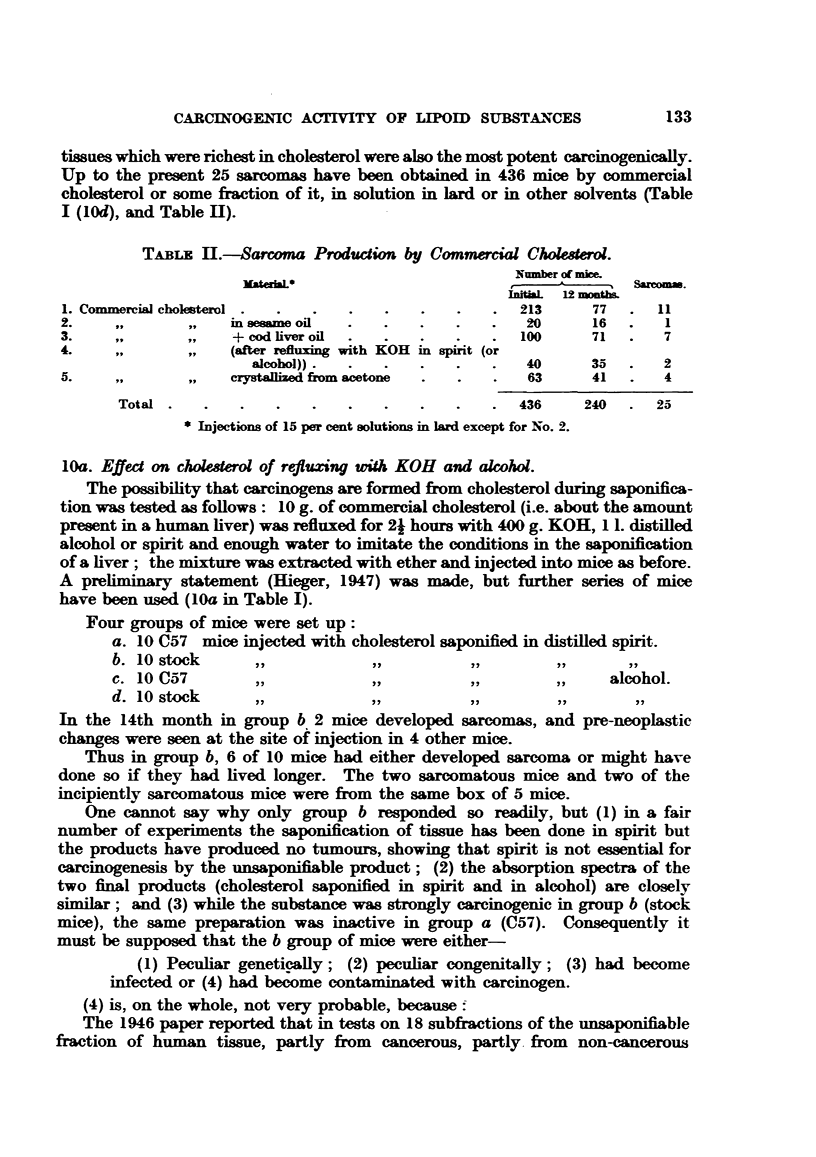

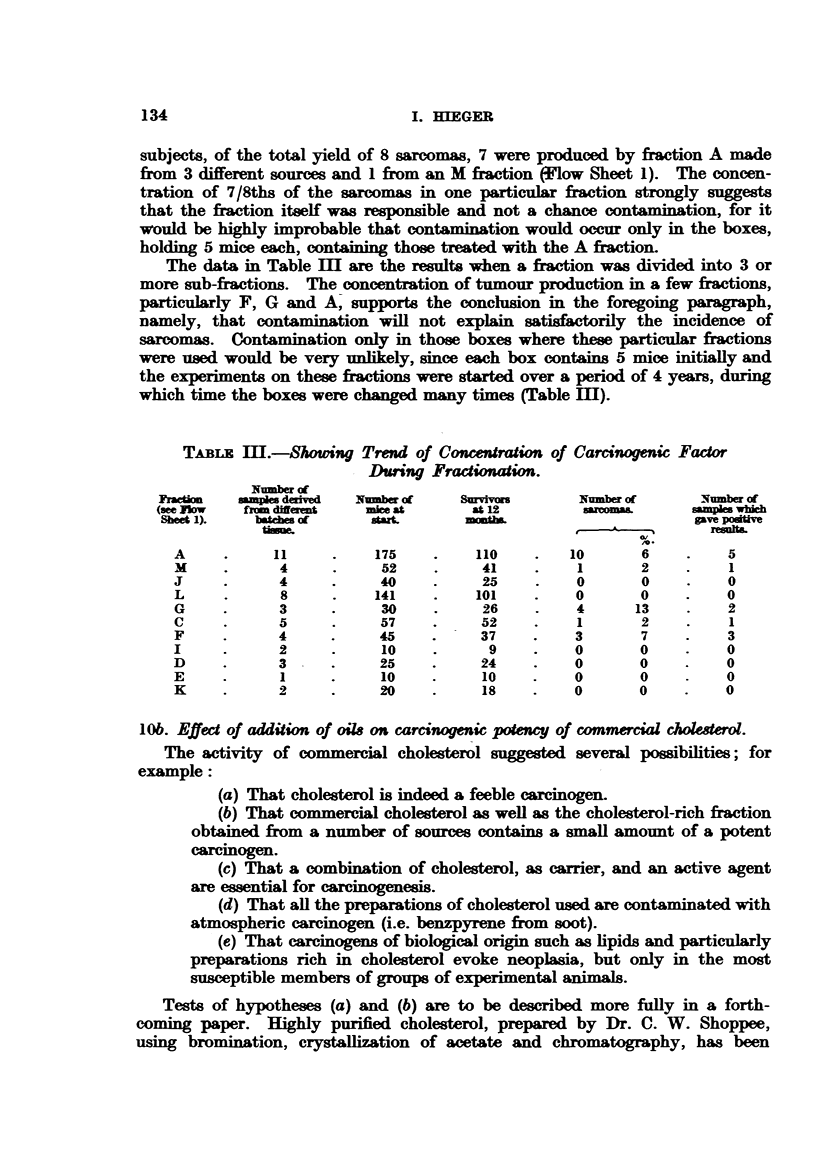

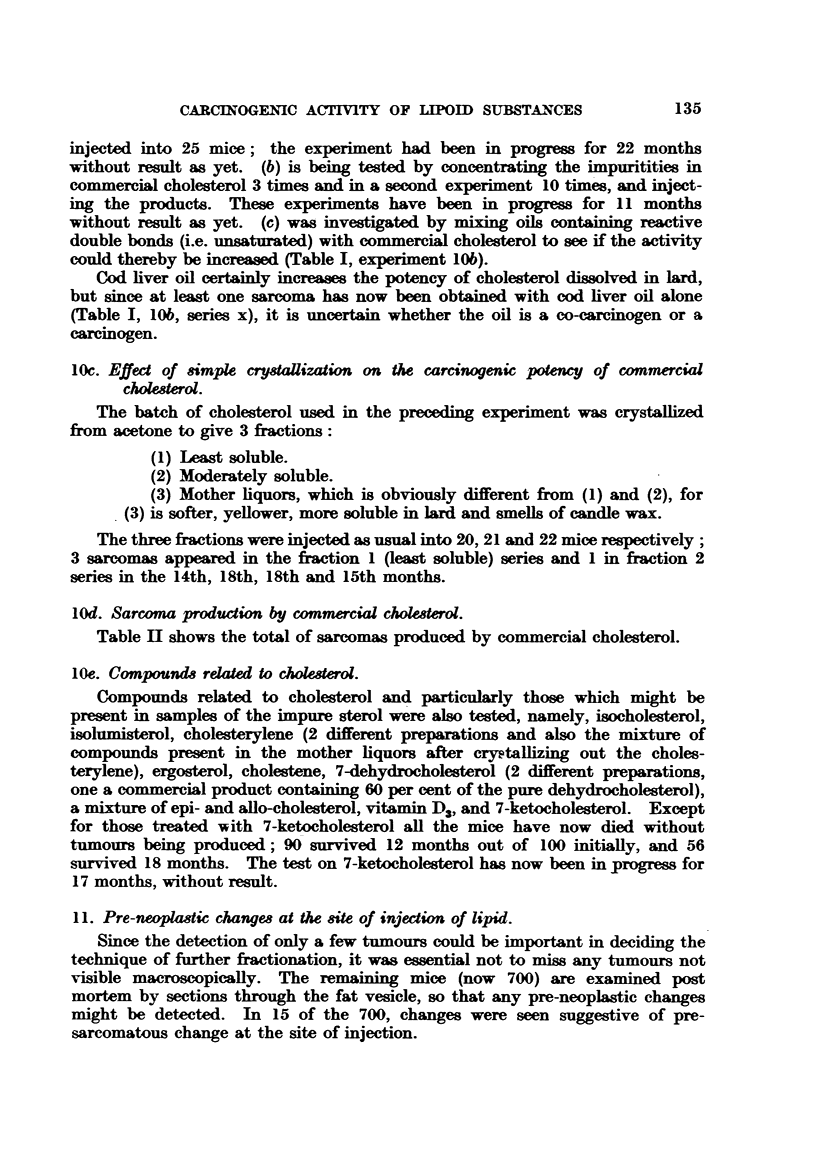

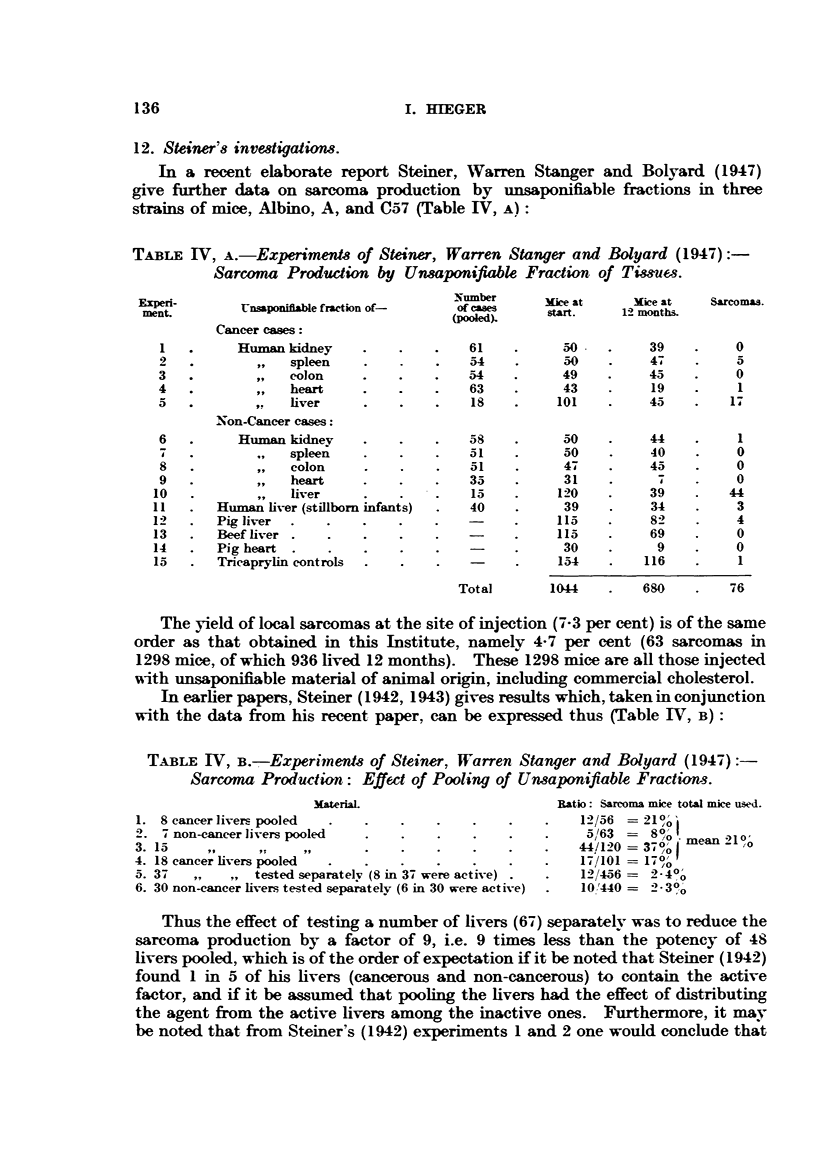

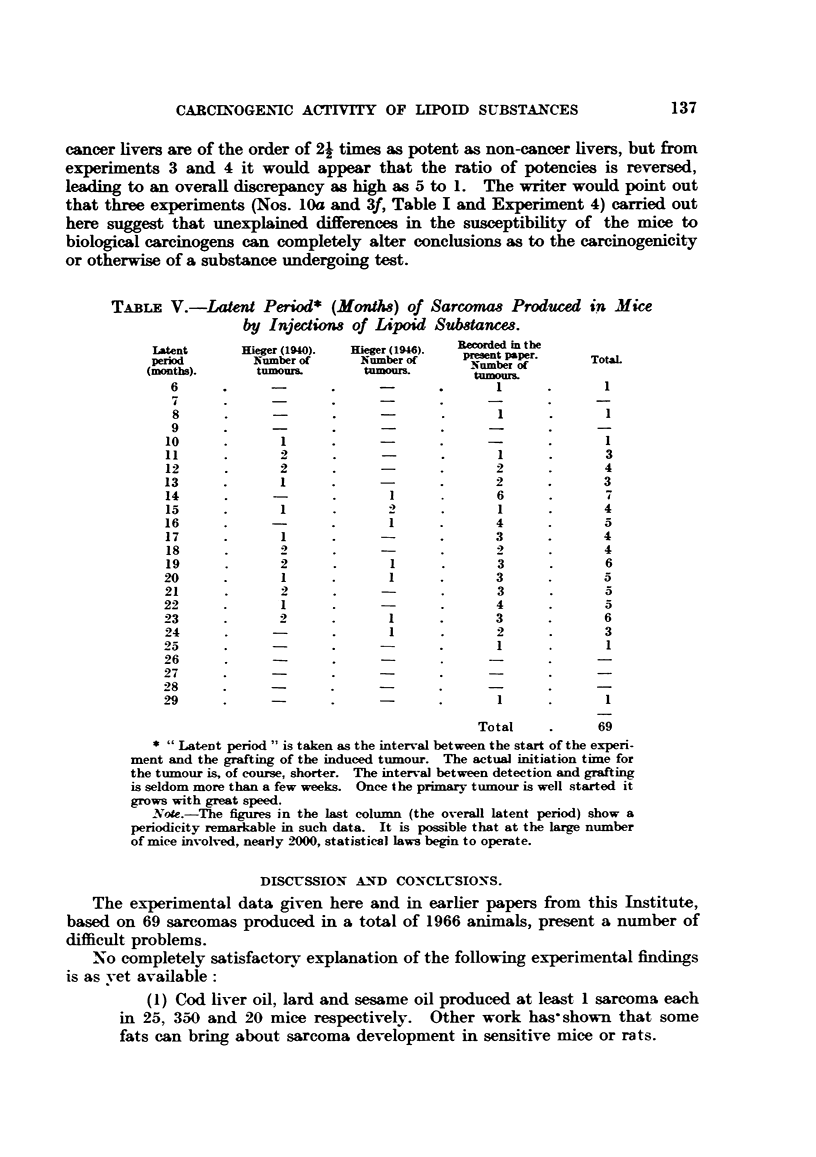

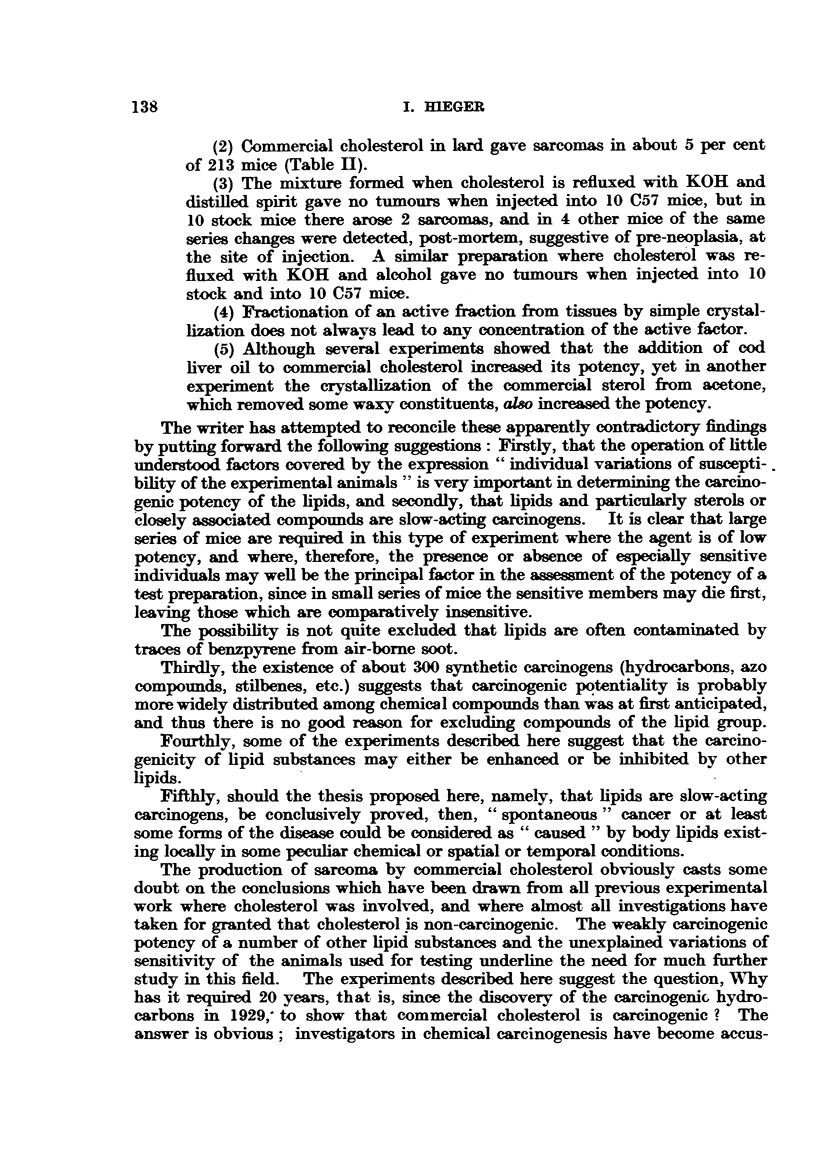

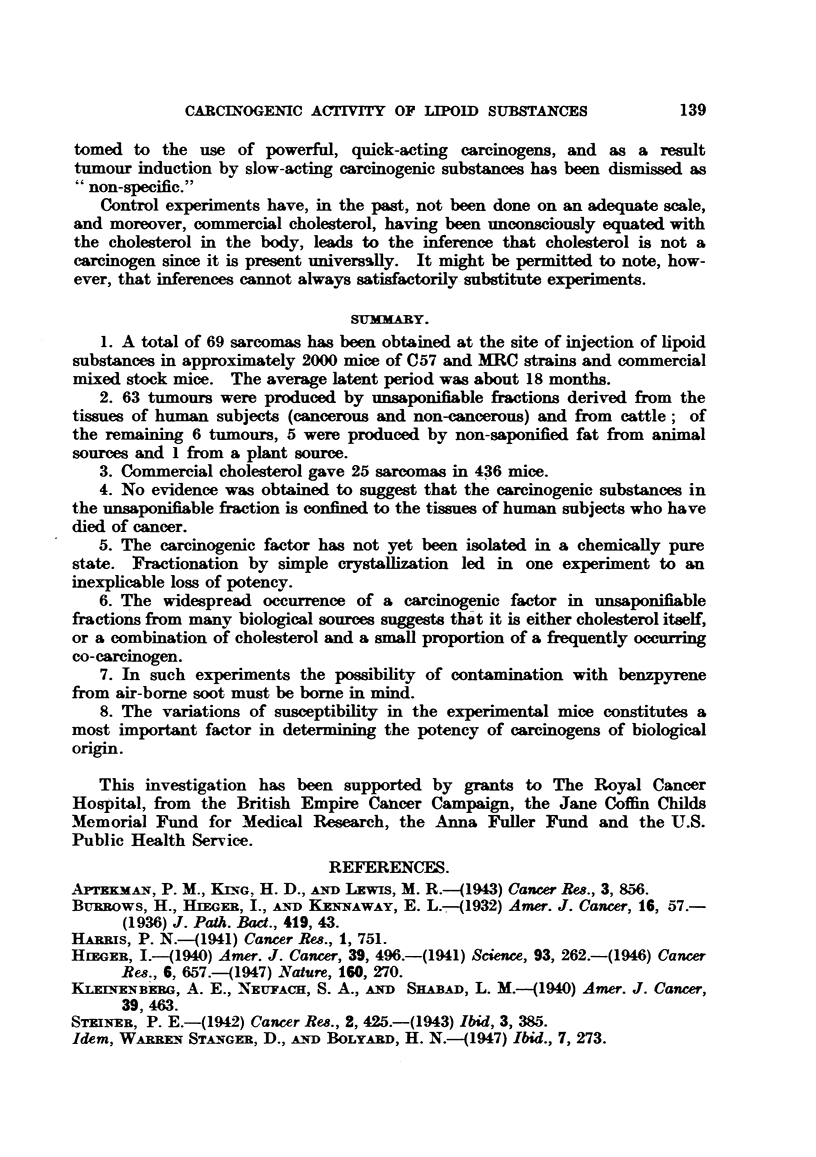

